# Investigation of Potential Therapeutic Effects of New Rapid-Acting Antidepressant Drugs (RAADs) Using Stress-Based Models of Depression

**DOI:** 10.3390/biom16091230

**Published:** 2026-08-24

**Authors:** Agnieszka Pałucha-Poniewiera

**Affiliations:** Department of Neurobiology, Maj Institute of Pharmacology, Polish Academy of Sciences, Smętna Street 12, 31-343 Kraków, Poland; nfpaluch@cyf-kr.edu.pl

**Keywords:** antidepressant drugs, chronic mild stress, forced swim test, ketamine, learned helplessness, RAAD, social defeat stress, tail suspension test

## Abstract

The use of animal models to study mental illnesses, such as depression, requires proper standardization and extensive expertise. Achieving good construct, face, and predictive validity in depression models is quite challenging. Currently, only a few environmental models, mostly based on chronic stress, and a limited number of genetic models fulfill these criteria. In the quest for new antidepressants, initial screening tests are employed as a preliminary step in research. While these tests do not always meet the requirements of a disease model, they are useful for the early identification of substances that may have antidepressant potential, paving the way for further studies based on established models. This approach to discovering antidepressants was originally designed for traditional medications, which typically act by modulating serotonergic, noradrenergic, and dopaminergic systems, and require multi-week administration to produce a therapeutic effect. In contrast, the new antidepressant ketamine offers rapid therapeutic effects following a single dose and exhibits distinctive behavioral outcomes in both screening tests and animal depression models. These effects have inspired a new model for the search for rapid-acting ketamine-like antidepressants. This review presents the behavioral effects of ketamine and discusses the methodologies used in the search for novel rapid-acting antidepressant drugs (RAADs).

## 1. Introduction

The requirement for long-term administration of antidepressant drugs (ADs) to induce a therapeutic effect is a well-known characteristic of these medications. This applies to tricyclics, monoamine oxidase (MAO) inhibitors, and the later-introduced selective serotonin reuptake inhibitors (SSRIs) and serotonin-norepinephrine reuptake inhibitors (SNRIs). Although these drugs work through different mechanisms and vary in effectiveness, patients typically experience symptom improvement only after several weeks. This delay is believed to be linked to adaptive changes in specific structures and pathways in the central nervous system (CNS) that occur due to prolonged exposure to these medications [1,2,3].

Research on the antidepressant effects of ketamine, an NMDA receptor antagonist originally used solely for anesthesia, has shown that its therapeutic benefits occur rapidly, typically within just a few hours to a couple of days after administering a single subanesthetic dose [4,5,6]. Notably, antidepressant effects have been observed in patients with treatment-resistant depression (TRD) [4,5,6]. Despite considerable controversy surrounding studies of ketamine as a treatment for depression, one of its enantiomers, *(S)*-ketamine, has been approved as an antidepressant, making it the first rapid-acting antidepressant drug (RAAD) available in clinical practice [7]. Independent studies have also demonstrated the antidepressant effects of scopolamine, an alkaloid with anticholinergic properties that is used to treat postoperative nausea and prevent motion sickness. Its antidepressant effects appear relatively rapidly compared to traditional ADs [8]. Furthermore, psilocybin, a naturally occurring psychedelic found in mushrooms of the genus Psilocybe, has also shown rapid and sustained antidepressant activity by acting on serotonergic receptors [9]. These findings suggest that substances with entirely different pharmacological profiles and intended medical uses can induce rapid antidepressant effects. However, a key question remains: do these substances share a common target that is responsible for their effects? Could this target become a therapeutic target for developing new, safer RAADs? Clarifying these issues has become one of the main research challenges in psychopharmacology over the past 20 years.

## 2. Investigating Antidepressant-like Effects in Rodents

### 2.1. Animal Models of Depression

Using animal models is the most effective way to study the therapeutic properties of pharmacologically active substances and their mechanisms of action. For potential drugs aimed at treating CNS diseases, animal models are essential for research due to the complexity and organization of this system. However, modeling mental illness in animals presents challenges, primarily due to the difficulty of replicating specific symptoms. Despite these challenges, researchers have developed several effective depression models that demonstrate good face and construct validity, as well as high predictive validity. Generally, these models are based on environmental, genetic, or biological modifications that relate closely to the risk factors for depression in humans.

The most commonly used animal models of depression that focus on environmental factors include:

- Chronic Mild Stress (CMS)/Chronic unpredictable mild stress (CUMS): This model involves exposing animals to various mild stressors in an unpredictable order over several weeks [10,11].
- Social Defeat Stress (SDS): In this model, animals are exposed to direct and/or indirect contact with an aggressor, which leads to the animal avoiding social interactions, thus weakening social connections [12].
- Learned Helplessness (LH): This model prevents the animal from escaping an electric stimulus after it has been conditioned to believe it cannot avoid this aversive stimulus [13].
- Chronic Restraint Stress (CRS): This model involves repeatedly placing the rodent in a tube that prevents it from escaping on a daily basis [14].
- Maternal Separation (MS): In this model, infant animals are separated from their mother for about three hours each day, during all or part of the lactation period [15].

**Figure 1:**
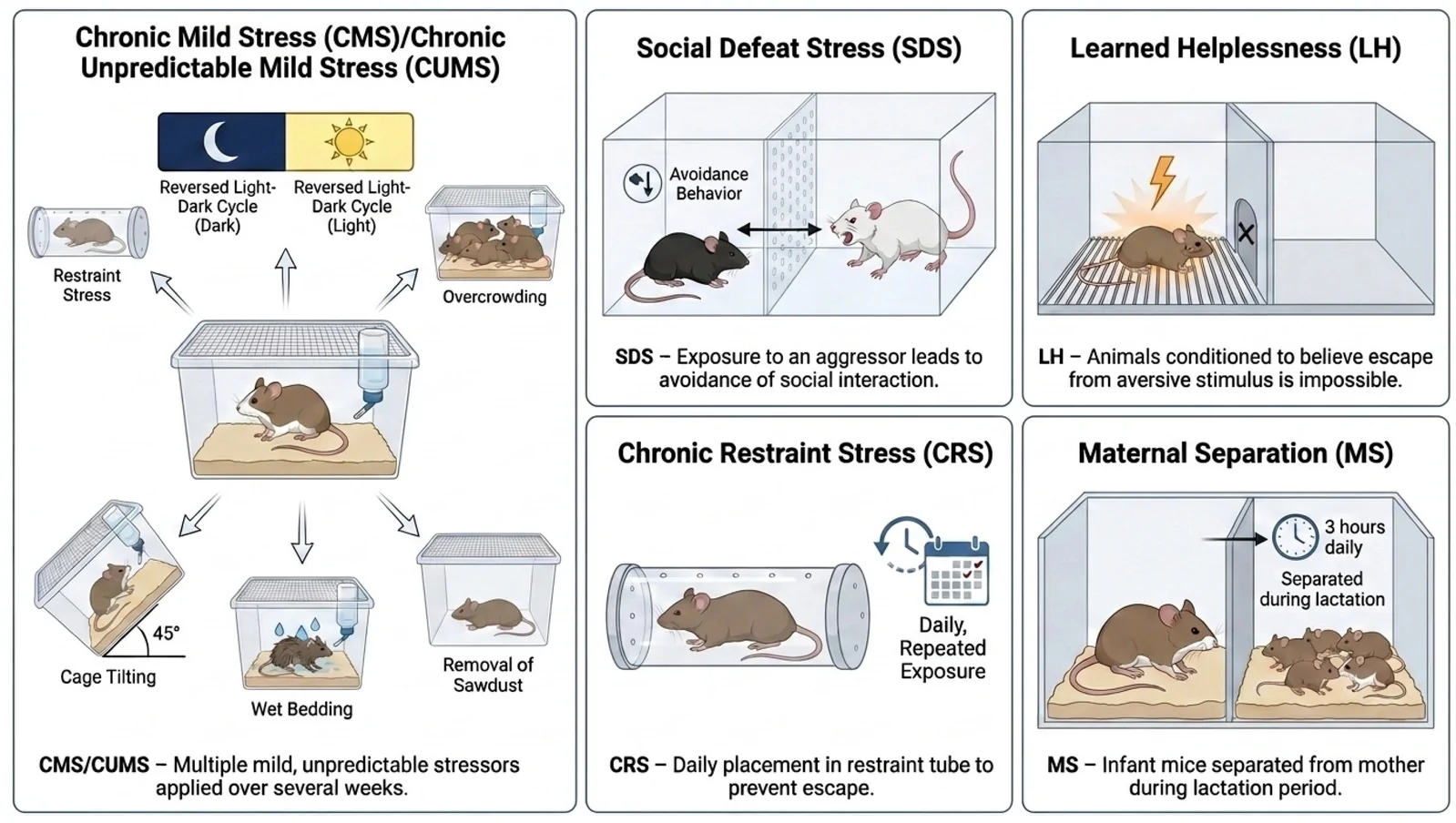
Environmental rodent models of depression. Modifications to environmental factors in rodent models of depression can lead to chronic stress and produce a depressive-like phenotype. The flowchart was conceptualized by the author and generated using FigureLabs (figurelabs.ai).

The discussed paradigms are shown schematically in Figure 1. In each of these models, prolonged exposure to repeated stressful stimuli results in behaviors that mirror human depression symptoms, such as anhedonia, reduced motivation and apathy. These behaviors can be reliably measured. Consequently, depression models can help identify potential antidepressant effects, which may manifest as improvements in stress-induced behavioral deficits. Importantly, the behavioral changes brought on by chronic stress tend to be relatively persistent, and traditional ADs typically require long-term administration to reverse them. This characteristic is advantageous for the model as it closely mimics the dynamics of therapeutic effects in patients [16]. It is important to note that the list of depression models used to search for new drugs also includes other procedures validated in specific laboratories, such as the chronic behavioral despair model [17].

Among the genetic models, the Flinders Sensitive Line (FSL) [18] and Wistar Kyoto rats [19] stand out as important models for depression and TRD, respectively. Notably, these rat strains were not originally developed to exhibit depressive symptoms; rather, the depressive phenotype in both cases was discovered by chance. However, both FSL and Wistar Kyoto rats exhibit neurobiological and behavioral changes that resemble depression, supporting their use as valuable models for specific variants of this disorder [18,19].

An example of a model based on biological modifications is the one aligned with the inflammatory theory of depression. This model involves administering lipopolysaccharide (LPS), a bacterial endotoxin [20,21]. LPS triggers the release of pro-inflammatory cytokines, causing a cascade of changes that, among other effects, results in decreased serotonergic transmission in the brain. This reduction in serotonergic activity is associated with symptoms of depression [22].

### 2.2. Depressive Phenotype in the Depression Model

**Figure 2:**
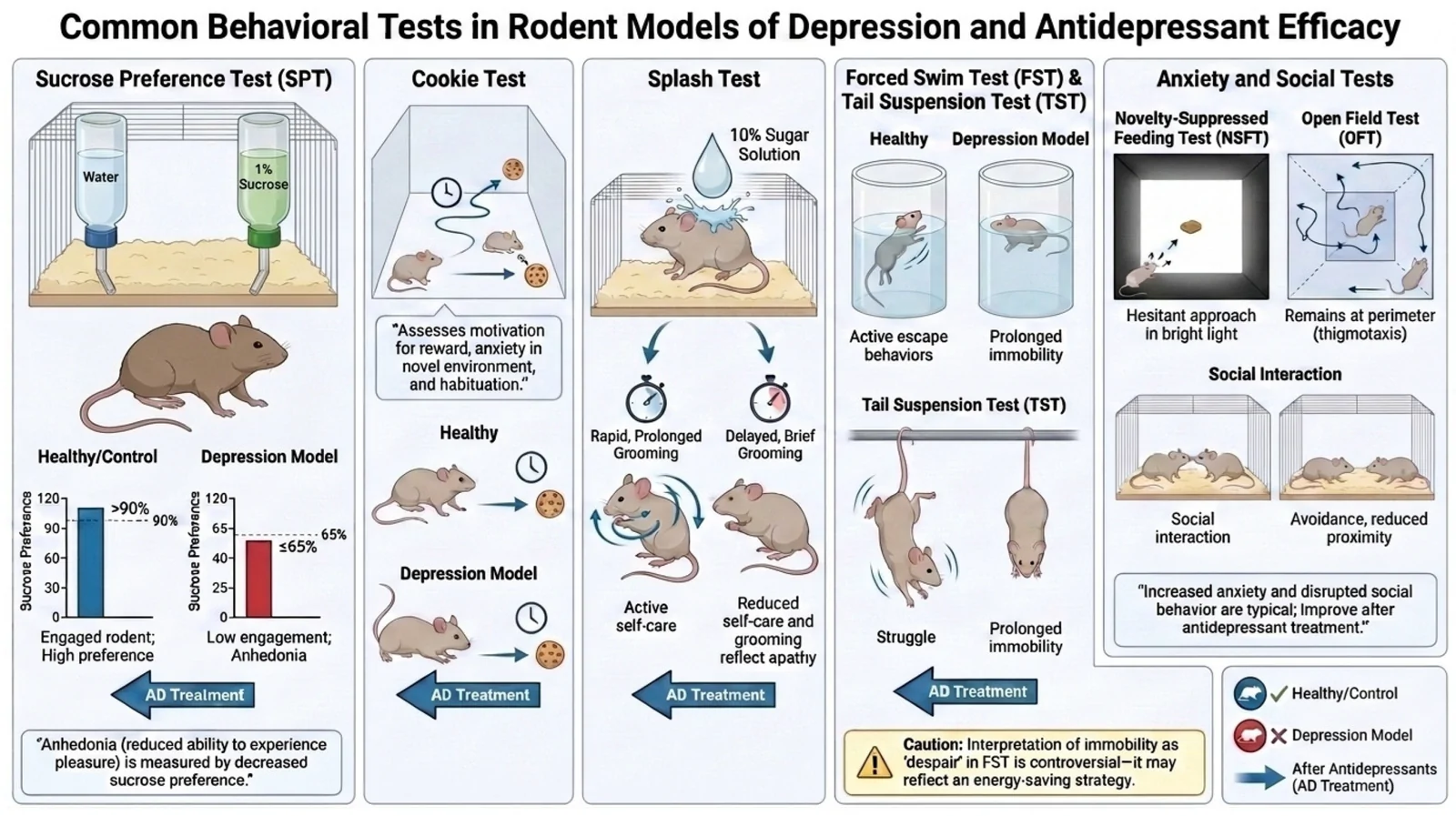
Behavioral tests to investigate the depressive phenotype and the potential antidepressant effects of tested substances in animal models of depression. The flowchart was conceptualized by the author and generated using FigureLabs (figurelabs.ai).

A depression model is valuable when it is associated with behavioral changes in animals that reflect the symptoms of depression and improve with the use of antidepressant medications. Behavioral tests to investigate the depressive phenotype and the potential antidepressant effects are schematically shown on Figure 2. One of the most common indicators of depression in animal models is a decreased preference for sucrose, which is considered a symptom of anhedonia, defined as a reduced ability to experience pleasure, a key symptom of depression [10,11,16]. The sucrose preference test (SPT) measures anhedonia by taking advantage of rodents’ natural tendency to prefer a 1% sucrose solution over water when given a choice [10,11,16]. In healthy animals, this preference can exceed 90% in favor of sucrose. However, in a depression model, this preference drops significantly, sometimes falling below 65% [23]. Classic ADs, when administered in a therapeutic regimen (i.e., over many weeks), gradually restore the preference for sucrose, thereby alleviating the anhedonia observed in the depression model [11,16].

An alternative to the SPT is the cookie test, which examines motivation for reward using a sweet cookie. An important advantage of the cookie test is that, apart from anhedonia, it also examines anxiety-like states while exploring novel environments as well as habituation to a novel environment, which are also linked to symptoms of depression [11].

Another symptom of the depressive phenotype that reflects depressive illness is a decreased focus on self-care, which may be linked to apathy [24]. Reduced self-grooming is often observed in the splash test, a procedure that measures the time spent grooming in the home cage after a 10% sugar solution is sprayed onto the animal’s back [11,25]. A healthy animal, feeling safe in its home environment, will typically start grooming to clean its fur. In contrast, animals experiencing depression tend to begin grooming later and spend less time doing so during the 5-min test. Similar to the SPT, classic antidepressants gradually restore the animals’ inclination to engage in self-grooming behavior and extend the duration of this activity [11,25].

Other behaviors that may indicate a depressive phenotype in animals include increased immobility time in the forced swim test (FST) [26,27] and the tail suspension test (TST) [28]. In the FST, a rat or mouse is placed in an inescapable cylinder of water for 5–6 min, while in the TST, a mouse is suspended by its tail for 6 min. Prolonged immobility in these tests is sometimes interpreted as a sign of despair and resignation, according to the developers of the tests [26,27,28]. However, it is important to note that the interpretation of animal immobility in the FST as a sign of despair is highly controversial and disputed by some in the scientific community [29]. Immobility during stress caused by an inescapable position may instead reflect an energy-saving strategy rather than resignation. Therefore, the FST should not be used as the sole indicator of a depressive phenotype; it should be considered as a potential supplement to other behavioral protocols.

The depressive phenotype can be further evaluated using tests that measure anxiety-related behaviors and disrupted social interactions, which often accompany symptoms of depression. Commonly used tests to assess anxiety behavior in depression models include the novelty-suppressed feeding test (NSFT) [30] and the open field test (OFT) [31]. In the NSFT, an animal that has been deprived of food must overcome its fear of entering a brightly lit, unfamiliar space to access food. In the OFT, anxious animals tend to avoid the central, well-lit area of an open field. In the tests mentioned above, as well as in other assessments of anxiety behavior, animals in the depression model typically exhibit increased anxiety behaviors. Conversely, those who are successfully treated with ADs show a reduction in anxiety behaviors. The same pattern is observed in social interactions, which are often negatively impacted in the depression model but improve significantly with prolonged administration of ADs [32].

### 2.3. Behavioral Screening Tests for Antidepressants

In addition to depression models, which must exhibit high face, construct, and predictive validity, behavioral screening tests are crucial in the search for new ADs. Screening tests are designed to quickly evaluate the antidepressant potential of a substance through a brief behavioral assessment. During this test, animals exhibit specific behaviors unique to antidepressants, allowing for a clear distinction from drugs with different action profiles, such as anxiolytics and neuroleptics. It is important to note that these screening tests are conducted on naive animals, rather than on models of disease. Tests that meet these criteria include the FST and TST. In practice, these tests serve as the initial assessments. Only substances that show promising antidepressant properties are advanced to testing using depression models.

The first, and still the most commonly used, screening test for identifying new ADs in rats and mice is the FST, which was described by Porsolt and colleagues in 1978 [26,27]. Another valuable screening test for searching for ADs is the TST in mice, developed in 1985 by Steru and colleagues [28]. It is important to note that potential antidepressant effects in these screening tests may be observed after a single or short-term administration of the medication, even though ADs typically take several weeks of regular use to become effective in patients.

Although the authors of the most commonly used screening test, the FST, referred to it as an “animal model of depression” [26,27], it is important to note that it does not fulfill most criteria for a disease model. This test measures the time of immobility during a short 5–6 min period of acute stress, but it does not accurately reflect the symptoms of depression, model the depressive phenotype, or mimic the dynamics of clinical drug action. The advantages of the FST include its simplicity, rapid results, ease of administration, and low cost. Most significantly, the FST specifically detects the effects of classic ADs, allowing them to be distinguished from other psychotropic drugs. The original studies by Porsolt et al. [26,27] demonstrated that various classes of antidepressants, such as tricyclics, MAO inhibitors, and atypical antidepressants, and electroconvulsive therapy (ECT) reduce immobility time in the FST while having no effect on locomotor activity or slightly reducing it. In contrast, other psychotropic medications do not exhibit this same pattern. For instance, psychostimulants like amphetamine and caffeine reduce immobility time in the FST at doses that increase locomotor activity. Anxiolytics, such as benzodiazepines, do not alter animal behavior in the FST, while neuroleptics like chlorpromazine can increase immobility time, which is correlated with a decrease in locomotor activity [26].

Moreover, Porsolt and colleagues made an important observation regarding the dosing regimen of antidepressants in the FST. They found that the most effective approach involved administering the drug either three times (at 24 h, 5 h, and 1 h before the test) or twice (at 24 h and 1 h before the test). A single dose given 24 h prior to the test did not reduce the immobility time of the animals being tested. Therefore, it is essential to administer the classic AD as a booster dose shortly (1 h) before the test to achieve a behavioral effect in the FST [26].

The FST was initially developed for rats [24] and was later adapted for mice [27] by the same research team. Research investigating various classes of psychotropic drugs has clearly shown that the FST procedure in mice is also selectively sensitive to antidepressant treatments.

The second commonly used screening test is the TST, which is mainly conducted on mice. In the TST, both typical and atypical ADs reduce the time that mice remain immobile when suspended by their tails for six minutes. Importantly, these substances do not affect or may even decrease locomotor activity [28]. Similar to the FST, this effect is specific to antidepressants. Other psychotropic drugs either fail to change immobility time in the TST or only reduce it at doses that enhance locomotor activity (such as psychostimulants) or increase immobility (such as benzodiazepines). Although the TST is used less frequently than the FST, it offers several advantages. First, it can effectively detect potential antidepressant effects after a single administration. Second, the test is straightforward, and the measurement of immobility time is objective, as it can utilize specialized measuring equipment. Additionally, it does not involve the use of stress combined with hypothermia.

It is important to keep in mind that both the FST and TST are screening tests. Therefore, their results should only be regarded as preliminary indications of a potential antidepressant’s effectiveness. To confirm the antidepressant properties of a substance, it must demonstrate a positive effect in a model of depression.

## 3. How Can We Study the Antidepressant-like Effects of RAADs in Depression Models?

The previously mentioned depression models and screening tests have been successfully used to investigate the mechanisms behind antidepressant effects and to search for new medications. However, it is important to note that these procedures were developed during a time when classic ADs were primarily utilized. These traditional medications mainly targeted the serotonergic, noradrenergic, and/or dopaminergic systems and required long-term administration to achieve therapeutic effects. Given this context, it is clear that applying the same tools and regimens to investigate the potential antidepressant effects of RAADs may be ineffective. RAADs exhibit different dynamics of action compared to classic ADs and likely operate through distinct cellular and molecular mechanisms.

The key to differentiating between classic ADs and RAADs in depression models is to compare the behavioral effects of typical ADs with those of the first and most well-known RAAD, ketamine. As previously mentioned, classic ADs need to be administered over several weeks to reverse the depressive phenotype observed in models, mirroring clinical practice. In contrast, the RAAD ketamine is expected to produce therapeutic effects in animal models of depression within 24 h to a few days after a single subanesthetic dose, similar to the regimen used in humans [4,5,6]. Following this design, studies have shown that ketamine at a subanesthetic dose (3–15 mg/kg) can reverse impaired sucrose preference in depression models and/or reduce self-grooming time, with effects lasting from 24 h to as long as two weeks after a single dose. Additionally, a single subanesthetic dose of ketamine has been proven to shorten prolonged immobility times in the FST and TST used in depression models (Table 1).

### 3.1. Chronic Mild Stress

Li and colleagues [33] demonstrated that a single dose of ketamine (10 mg/kg) improved sucrose preference in the SPT in rats subjected to the CMS model of depression. The anhedonia-relieving effect lasted for up to 7 days following a single administration. The same study also found that a single dose of ketamine (10 mg/kg) reduced the latency to feed in the NSFT to levels observed in non-stressed control rats, indicating that ketamine influences stress-induced anxiogenic behaviors [33]. Additionally, another report showed the effectiveness of ketamine at a dose of 10 mg/kg in alleviating long-term stress-induced symptoms of anhedonia in the SPT and despair in the FST in rats at 72 h after the single administration [34]. Furthermore, a study by Maciel and colleagues found that a single dose of 15 mg/kg of ketamine was effective in the FST in rats at two weeks post-treatment [35].

The reversal of CMS effects by a single dose of ketamine has also been observed in mice. For instance, a single dose of 3 mg/kg of ketamine reversed the decrease in sucrose preference caused by 4 weeks of CMS in C57BL/6 mice, measured 24 h after injection [36]. Additionally, ketamine demonstrated beneficial effects in the FST and the NSFT in this depression model [36]. A similar effect was seen in C57BL/6J mice subjected to a 5-week CMS procedure, where a single dose of 10 mg/kg of ketamine reversed CMS-induced anhedonia in the SPT, lasting from 24 h to 8 days after administration [37]. It also reversed CMS-induced prolongation of immobility in both the FST and TST conducted 2 days after drug administration [37]. Another study using the CMS model with C57BL/6J mice found that a single dose of ketamine exhibited antidepressant-like activity in the TST and FST. Additionally, it affected sucrose preference after 3 days of administration at the same dose, but not at 3 mg/kg [38]. A higher yet still subeffective single dose of ketamine (30 mg/kg) reduced CMS-induced anhedonia in the SPT from 2 h to 2 days post-administration. In contrast, the SSRI fluoxetine, administered as a single dose of 15 mg/kg, did not impact this effect [39]. The antidepressant-like effect of this ketamine dose in the CMS model of depression was confirmed using the FST, TST, and NSFT. Notably, ketamine was found to reverse the CMS-induced increase in immobility in both the FST and TST for up to 5 days following a single administration [39].

Interestingly, a comparative study of the antidepressant-like properties of ketamine enantiomers, *(S)*-ketamine and *(R)*-ketamine, was conducted using the CMS model in C57BL/6J mice. The results showed that a single dose of *(S)*-ketamine (10 mg/kg) eliminated the effects of chronic stress in the splash test 24 to 72 h after administration and in the SPT 24 h post-injection. In contrast, a single dose of *(R)*-ketamine (10 mg/kg) demonstrated effects from 24 h up to 7 days after administration in both tests. These findings indicate that both ketamine enantiomers exhibit rapid and sustained anti-apathetic and anti-anhedonic effects, but the effects of *(R)*-ketamine appear to be more lasting [40].

A study comparing the effects of ketamine in male and female C57BL/6J mice provided new, significant data on the reversal of CMS effects. At a dose of 10 mg/kg, ketamine induced antidepressant-like effects in the FST both 24 h and 7 days after administration, but this effect was observed only in males. In females, the antidepressant effect was evident only 24 h after treatment. Additionally, in the splash test, an anti-apathy-like effect was seen 5 days after administration, but again, this was only in males. Conversely, in the OFT, ketamine’s anxiolytic effects were observed solely in females [41].

On the other hand, some reports indicate that single administrations of ketamine have no effect in the CMS model, both in rats and mice. For example, a study found that a dose of 15 mg/kg of ketamine did not alter sucrose preference in the CMS model in rats. However, this dose did reverse the CMS-induced increase in adrenal gland weight, promote the regain of body weight, and normalize circulating levels of corticosterone and ACTH [42]. In mice, both female and male subjects subjected to CMS also showed no change in sucrose preference at six days post-treatment following a single administration of ketamine at a dose of 10 mg/kg. Nevertheless, the same experiment revealed antidepressant-like effects of ketamine in male mice during the splash test and the FST [41].

### 3.2. Social Defeat Stress

A rapid and sustained antidepressant-like effect from a single dose of ketamine was also observed in the SDS model of depression. The effects of ketamine in the mouse SDS model demonstrated that a single dose (10 mg/kg) effectively reversed the stress-induced decrease in sucrose preference at one, three, and seven days after administration [43]. A study conducted in a different laboratory revealed that administering ketamine twice (10 mg/kg) to C57BL/6J mice not only resulted in a prolonged antidepressant effect observed in the SPT and FST but also significantly enhanced social interactions that were impaired in the SDS model [44].

The rapid and sustained antidepressant-like effects of ketamine and both of its enantiomers in the SDS model have been confirmed in studies that used intranasal administration, which is similar to the method used in human treatments. Beneficial effects were observed for all substances in the FST 24 h after administration and in the SPT two days after administration. Furthermore, *(R)*-ketamine demonstrated a reversal of SDS-induced anhedonia for up to 7 days following a single intranasal treatment [45].

Research involving female C57BL/6J mice confirmed that a single dose of ketamine (20 mg/kg) can reverse the SDS-induced behavioral impairments. Notably, these effects were only seen in the phenotype that was susceptible to SDS [46].

Importantly, the research shows that the SDS model can distinguish RAADs from classically acting ADs. For example, a single dose (10 mg/kg) of *(S)*-citalopram, an SSRI, did not show antidepressant-like effects in the SDS model, even though the same experimental design that identified the effects of a single dose of ketamine was utilized [47]. Also, recent research by Shintani and colleagues [48] indicated that another SSRI, fluoxetine, demonstrated its antianhedonic effect in the SDS model only after 14 days of consistent administration at a dosage of 20 mg/kg per day, but not after a single treatment. In contrast, the effects of ketamine at a dosage of 20 mg/kg were noticeable soon after a single administration and lasted for up to 14 days [48]. A recent study by Domżalska and colleagues [49] provides important comparative data on the use of the SDS model for detecting rapid antidepressant effects. According to their findings, fluoxetine requires two weeks of administration (20 mg/kg/day) to eliminate the reduced social preference observed in the SDS model. In contrast, ketamine (10 mg/kg) and psilocybin (10 mg/kg), both of which induce rapid antidepressant effects in humans, show antidepressant-like effects in the SDS model within 24 h, as well as at 7 and 14 days after a single treatment [49].

Research conducted on rats did not support the effectiveness of a single dose of ketamine (20 mg/kg) in alleviating depression-like symptoms in the SDS model. This suggests that the effectiveness of RAAD detection may be restricted to the SDS model used in mice [50].

### 3.3. Learned Helplessness

The effects of single-dose ketamine have also been examined using the learned helplessness (LH) model of depression. In this model, animals that undergo the LH procedure typically respond to traditional ADs treatments only after extended use. An initial study exploring the rapid antidepressant mechanism of ketamine in Sprague-Dawley rats found that a single administration of ketamine (10 mg/kg) significantly reduced the number of escape failures in LH rats [51]. This effect has been confirmed in further studies using the LH model with both Sprague-Dawley and Wistar Kyoto rats, the latter being considered a model for TRD [52]. Notably, one of the enantiomers of ketamine, *(R)*-ketamine, demonstrated rapid antidepressant-like effects in a rat LH model after a single administration, with significant results observed both 24 h and 5 days post-treatment [53]. In contrast, a single dose of the classic AD desipramine did not produce antidepressant-like effects in the LH model in rats, as evidenced by the results in both the SPT and the FST [54].

In mice, the rapid effect of ketamine in the LH model of depression was demonstrated for the first time by Autry and colleagues [36], who used a single dose of 3 mg/kg. Subsequent studies have confirmed that a single injection of low-dose ketamine significantly reduces both the number of escape failures and the latency to escape 24 h after treatment [55,56]. This effect persists even up to 7 days after a single injection [57]. Additionally, in the LH model using Swiss mice, ketamine at doses of 10 or 30 mg/kg was effective in relieving anhedonia, as demonstrated by improvements in the SPT, and in prolonging immobility time in the TST, five days after administration [58].

### 3.4. Other Depression Models

The antidepressant-like effect of a single dose of ketamine (10 mg/kg) was demonstrated in the CRS model of depression in Wistar Kyoto rats 24 h after drug administration. Behavioral effects were observed in the SPT and TST [59]. Ketamine has also been shown to enhance the RCS-impaired sucrose preference in C57BL/6J mice [60]. In this study, ketamine was administered at a dosage of 10 mg/kg for three consecutive days, and the SPT was conducted 24 h after the final administration. Additionally, the effects of ketamine were found to be comparable to those produced by a 14-day regimen of daily administration of the tricyclic antidepressant imipramine (10 mg/kg/day) [60]. Another study in C57BL/6J mice using the CRS model found that a single dose of ketamine (10 mg/kg) administered 24 h earlier was enough to affect animal behavior in the TST and FST [61]. Additionally, low-dose ketamine treatment reversed social dysfunction and depression-like behaviors induced by CRS in male and female C57BL/6 mice, with doses of 10 mg/kg for males and 5 mg/kg for females [62].

In a study utilizing the MS model of depression in rats, the effectiveness of a single dose of ketamine (15 mg/kg) was demonstrated in the FST. This effect was found to be comparable to that of a tricyclic AD, amitriptyline (10 mg/kg), which was administered chronically over three weeks [35]. Additionally, a single dose of ketamine (30 mg/kg) was shown to be effective in a mouse model of postpartum depression. Specifically, 24 h after a single injection, ketamine not only increased self-grooming behavior in the splash test but also led to an increase in immobility time in the TST, suggesting a rapid counteraction of postpartum stress-induced behaviors [63].

Notably, the swift effectiveness of ketamine (10 mg/kg) in alleviating CMS-induced anhedonia was shown in Wistar Kyoto rats, a strain that is resistant to long-term treatment with traditional ADs and is considered a genetic model for TRD [19]. In another genetic model of depression using FSL rats, a single dose of ketamine (15 mg/kg) produced a sustained antidepressant-like effect, as measured by the FST. In contrast, fluoxetine (10 mg/kg) and vortioxetine (10 mg/kg) did not demonstrate any such effect in FSL rats [19].

**Table 1:** Antidepressant-like effects of a single ketamine administration in animal models of depression as a paradigm of RAAD action (a description of the results contained in the table can be found in Section 3). The table presents results from CMS and SDS studies assessing the effects of a single subanesthetic dose of ketamine or its enantiomers from 24 h to 2 weeks after administration, depending on the methodology used. In the LH model of depression, shorter times after ketamine administration were taken into account (30 min) due to the methodological specificity of the test. Compounds were administered intraperitoneally, except for the study involving intranasal administration, designated as ^. The table includes only the results obtained in males. CMS—chronic mild stress; CRS—chronic restraint stress; CUMS—chronic unpredictable mild stress; EPM—elevated plus maze; FST—forced swim test; LH—learned helplessness; MS—maternal separation; NSFT—novelty-suppressed feeding test; OFT—open field test; SDS—social defeat stress; SPT—sucrose preference test; TST—tail suspension test.

Depression Model	Drug	Dosage	Behavioral Test	Post-Treatment Effect	Reference
CMS/CUMS in rats	Ketamine	10 mg/kg	SPT	24 h–7 days	[30]
	Ketamine	10 mg/kg	SPT	72 h	[31]
	Ketamine	10 mg/kg	NSFT	48 h	[30]
	Ketamine	10 mg/kg	FST	72 h	[31]
	Ketamine	15 mg/kg	FST	8 days	[32]
	Ketamine	15 mg/kg	SPT	No effect	[39]
CMS/CUMS in mice	Ketamine	3 mg/kg	SPT	30 min	[33]
	Ketamine	3 mg/kg	FST	24 h	[33]
	Ketamine	3 mg/kg	NSFT	48 h	[33]
	Ketamine	10 mg/kg	SPT	24 h–8 days	[34]
	Ketamine	10 mg/kg	SPT	No effect	[38]
	Ketamine	10 mg/kg	FST	48 h	[34]
	Ketamine	10 mg/kg	FST	24 h–7 days	[38]
	Ketamine	10 mg/kg	FST	24 h	[35]
	Ketamine	10 mg/kg	TST	48 h	[34]
	Ketamine	10 mg/kg	TST	24 h	[35]
	Ketamine	10 mg/kg	Splash test	5 days	[38]
	Ketamine	30 mg/kg	SPT	2 h–2 days	[36]
	Ketamine	30 mg/kg	TST	24 h–5 days	[36]
	Ketamine	30 mg/kg	FST	24 h–5 days	[36]
	Ketamine	30 mg/kg	NSFT	48 h	[36]
	*(S)*-Ketamine	10 mg/kg	Splash test	24 h–3 days	[37]
	*(S)*-Ketamine	10 mg/kg	SPT	24 h	[37]
	*(R)*-Ketamine	10 mg/kg	Splash test	24 h–7 days	[37]
	*(R)*-Ketamine	10 mg/kg	SPT	24 h–7 days	[37]
SDS in rats	Ketamine	20 mg/kg	SPT	No effects	[48]
SDS in mice	Ketamine	10 mg/kg	SPT	24 h–7 days	[41]
	Ketamine	10 mg/kg ^	SPT	48 h	[43]
	Ketamine	10 mg/kg ^	FST	24 h	[43]
	Ketamine	10 mg/kg	Social preference	24 h–14 days	[47]
	Ketamine × 2	10 mg/kg	SPT	24 h	[42]
	Ketamine × 2	10 mg/kg	FST	24 h	[42]
	Ketamine × 2	10 mg/kg	Social interaction	24 h	[42]
	Ketamine	20 mg/kg	SPT	1–14 days	[46]
	*(S)*-Ketamine	10 mg/kg	SPT	24 h–6 days	[40]
	*(S)*-Ketamine	10 mg/kg ^	SPT	48 h	[43]
	*(S)*-Ketamine	10 mg/kg	TST	2–7 days	[40]
	*(S)*-Ketamine	10 mg/kg	FST	2–7 day	[40]
	*(S)*-Ketamine	10 mg/kg ^	FST	24 h	[43]
	*(R)*-Ketamine	10 mg/kg	SPT	24 h–6 days	[40]
	*(R)*-Ketamine	10 mg/kg ^	SPT	2–7 days	[43]
	*(R)*-Ketamine	10 mg/kg	TST	2–7 days	[40]
	*(R)*-Ketamine	10 mg/kg	FST	2–7 days	[40]
	*(R)*-Ketamine	10 mg/kg ^	FST	24 h	[43]
LH in rats	Ketamine × 4	5 mg/kg	Escape failures	24 h	[50]
	Ketamine	10 mg/kg	Escape failures	24 h	[49]
	*(R)*-Ketamine	20 mg/kg	Escape failures	24 h–5 days	[51]
LH in mice	Ketamine	3 mg/kg	Latency to escape	30 min	[33]
	Ketamine	10 mg/kg	Escape failures	24 h	[53,54]
	Ketamine	10 mg/kg	Latency to escape	24h	[53,54]
	Ketamine	10 mg/kg	Escape failures	24 h–7 days	[55]
	*(S)*-Ketamine	10 mg/kg	Latency to escape	24 h	[56]
	*(S)*-Ketamine	10 mg/kg	Failure to escape	24 h	[56]
	*(S)*-Ketamine	20 mg/kg	Latency to escape	No effect	[40]
	*(S)*-Ketamine	20 mg/kg	Failure to escape	No effect	[40]
	*(R)*-Ketamine	20 mg/kg	Latency to escape	5 days	[40]
	*(R)*-Ketamine	20 mg/kg	Failure to escape	5 days	[40]
CRS in mice	Ketamine	10 mg/kg	TST	24 h	[59,60]
	Ketamine	10 mg/kg	FST	24 h	[59]
	Ketamine × 3	10 mg/kg	SPT	24 h	[58]
MS	Ketamine	15 mg/kg	FST	8 days	[32]
Postpartum depression	Ketamine	30 mg/kg	Splash test	24 h	[61]
	Ketamine	30 mg/kg	TST	24 h	[61]
Wistar Kyoto	Ketamine × 8	10 mg/kg	SPT	24 h	[62]
	Ketamine × 8	10 mg/kg	EPM	24 h	[62]
Wistar Kyoto + CRS	Ketamine	10 mg/kg	SPT	24 h	[57]
	Ketamine	10 mg/kg	TST	24 h	[57]
	Ketamine	10 mg/kg	OFT	24 h	[57]

## 4. Using Screening Tests to Assess Antidepressant-like Effects of RAADs

Employing screening tests such as the FST or TST in regimens designed for traditional ADs to evaluate the antidepressant-like effects of RAADs is not justified. This is because these tests typically measure therapeutic potential 30 to 60 min after a single or short-term administration of an ADs. In clinical practice, however, these medications generally require weeks of consistent administration to show efficacy. Therefore, while a reduction in immobility observed in the FST or TST soon after ketamine administration might suggest its antidepressant activity, this result would not adequately capture its rapid effects. Moreover, caution is warranted when examining the short-term effects of ketamine (30–60 min post-administration) as it may impact locomotor activity, induce dissociative effects, and alter perception—factors that are critical for accurately assessing mood in humans.

The potential to differentiate between classic ADs and RAADs using screening tests emerged when the molecular and structural mechanisms underlying the antidepressant effects of ketamine were proposed. Preclinical studies have demonstrated that the rapid antidepressant effect of ketamine is associated with neuroplastic changes that can be detected as soon as 24 h after a single administration. As a result, researchers chose to conduct screening tests 24 h post-injection of ketamine. Numerous studies have confirmed that ketamine induces antidepressant-like effects in these tests between 24 h and several days after a single administration, unlike classic ADs, which do not reduce immobility time within 24 h after injection. Notably, research using the FST in rats revealed that this effect is specific to subanesthetic doses of ketamine (10 mg/kg), which produces an antidepressant-like effect, while anesthetic doses (80 mg/kg) do not have such effects [51].

The antidepressant-like effect of ketamine (10 mg/kg) 24 h after injection was confirmed in C57BL/6J mice, both in the FST and the TST [37]. A similar effect was noted in CD-1 mice during the FST by Zanos and colleagues [57], who also reported that fluoxetine (20 mg/kg) did not produce a sustained antidepressant-like effect under the same testing conditions [57]. In ICR mice, a single administration of ketamine (10 mg/kg) led to antidepressant-like effects in the TST that lasted up to three days post-injection. In contrast, desipramine (3 mg/kg), used as a reference drug, only demonstrated activity in the TST for 30 min after treatment and did not result in prolonged effects [64].

Another study conducted on mice demonstrated that a lower dose of ketamine (3 mg/kg) also reduced immobility time in the FST 24 h after administration. This effect lasted for up to a week following a single injection. In contrast, other NMDA antagonists, including NMDA channel blockers, did not produce similar effects [36]. A comparative study assessing the rapid antidepressant-like effect of ketamine in both male and female C57BL/6J mice revealed that, in males, ketamine significantly reduced immobility time in the FST 24 h after treatment at a dose of 10 mg/kg (lower doses were ineffective). In females, however, the antidepressant-like effect was observed at a lower dose of 5 mg/kg 24 h post-administration [41].

**Figure 3:**
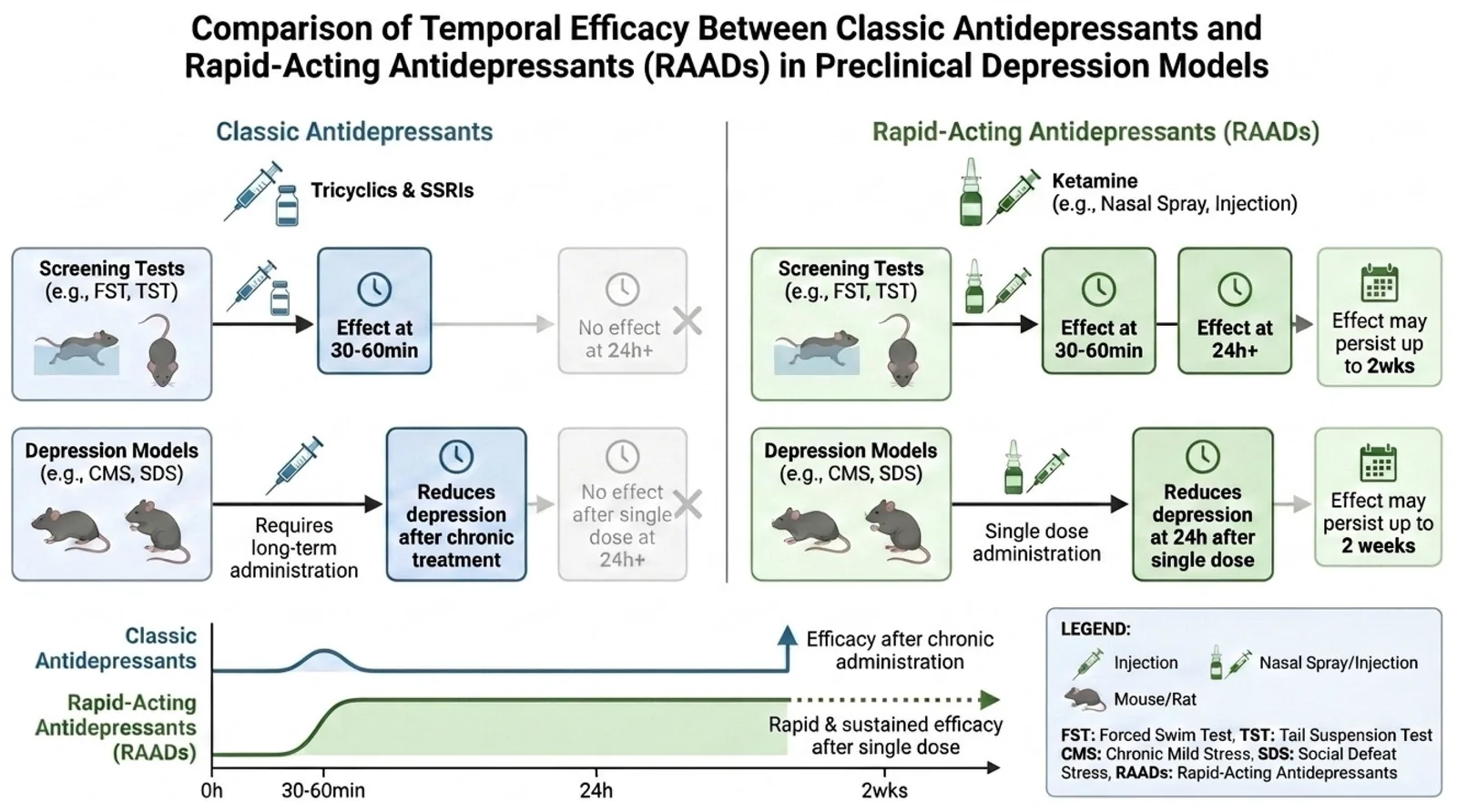
Summary of the most important differences between the action of classic ADs and RAADs in screening tests and animal models of depression. The diagram refers to the data described in paragraphs 3 and 4. The flowchart was conceptualized by the author and generated using FigureLabs (figurelabs.ai).

A comparison of the antidepressant-like effects of ADs and RAADs in animal models of depression and screening tests is shown in Figure 3.

## 5. Molecular Mechanism of RAADs

The distinct behavioral effects of classical ADs and RAADs stem from their different mechanisms of action at the molecular and cellular levels. The therapeutic effects of traditional ADs, which require long-term use, are associated with adaptive changes primarily involving the serotonergic and noradrenergic systems [1,2,3]. In contrast, research into the mechanisms behind ketamine’s rapid antidepressant effects has produced extensive, though inconsistent, evidence regarding the involvement of various molecular and cellular processes across different brain regions. A common finding among these studies is the swift enhancement of neuroplasticity in brain areas that regulate emotional and motivational processes, such as the prefrontal cortex [65], anterior cingulate cortex [66], hippocampus [67], and habenula [68]. This increase in neuroplasticity results in a higher number of dendritic spines and a corresponding increase in synaptic connections in regions critical to depression [69]. The mechanisms that promote enhanced neuroplasticity may involve the regulation of several factors related to protein translation, such as mTOR kinase [51] and eEF2 kinase [36], as well as other elements necessary for synapse formation, like brain-derived neurotrophic factor (BDNF) [70]. Additionally, changes in the levels of proteins that form the structural and functional framework of active synapses, such as postsynaptic density protein 95 (PSD-95), may reflect the intensity of neuroplastic processes [51]. Finally, glutamate receptors, particularly AMPA receptor subunits (GluA1–4), are important to consider, as their levels are directly linked to synaptic activity and synapse efficiency [71]. This aspect should also be included in studies addressing neuroplastic changes. Although a detailed discussion of the role of RAADs in regulating neuroplasticity is beyond the scope of this review, which focuses on behavioral aspects, it is important to highlight that including neuroplasticity in evaluating the antidepressant effects of new potential RAADs is essential and should complement behavioral assessments.

## 6. Conclusions

Investigating the therapeutic potential of new RAADs requires a specialized approach. This involves using a specific schedule for screening tests, followed by behavioral testing in animal models of depression. In the screening tests, it is essential to assess both the acute effects of the drug, such as observations made 30 to 60 min after administration, which may suggest possible antidepressant activity and the delayed effects. The latter can be evaluated 24 h after administration, and potentially up to a week or longer, to determine if the drug may work through mechanisms characteristic of RAADs. Behavioral studies using a depression model require careful assessment of animal behaviors in tests designed to reflect symptoms of depressive illness, such as anhedonia, decreased motivation, or apathy. These behaviors should be evaluated 24 h after a single or short-term administration of the test drug. This approach helps distinguish RAADs from traditional ADs, which typically produce therapeutic effects only after repeated administrations. Furthermore, it is necessary to investigate the mechanism of action of the tested drug and evaluate its impact on neuroplastic processes within brain regions involved in emotion and motivation.

## Data Availability

No new data were created or analyzed in this study. Data sharing is not applicable to this article.

## Abbreviations

The following abbreviations are used in this manuscript:

AD	Antidepressant Drug
ACTH	Adrenocorticotropic Hormone
AMPA	Alpha-Amino-3-Hydroxy-5-Methyl-4-Isoxazolepropionic Acid
BDNF	Brain-Derived Neurotrophic Factor
CMS	Chronic Mild Stress
CNS	Central Nervous System
CRS	Chronic Restraint Stress
CUMS	Chronic Unpredictable Mild Stress
ECT	Electroconvulsive Therapy
eEF2	Eukaryotic Translation Elongation Factor 2
FSL	Flinders Sensitive Line
FST	Forced Swim Test
LH	Learned Helplessness
LPS	Lipopolysaccharide
MAO	Monoamine Oxidase
MS	Maternal Separation
mTOR	Mammalian Target of Rapamycin
NMDA	N-Methyl-D-Aspartic Acid
NSFT	Novelty-Suppressed Feeding Test
OFT	Open Field Test
PSD-95	Postsynaptic Density Protein 95
RAAD	Rapid-Acting Antidepressant Drug
SDS	Social Defeat Stress
SNRI	Serotonin-Norepinephrine Reuptake Inhibitors
SPT	Sucrose Preference Test
SSRI	Selective Serotonin Reuptake Inhibitor
TRD	Treatment-Resistant Depression
TST	Tail Suspension Test
